# DIFFERENCES IN COGNITIVE DECLINE DUE TO ALZHEIMER’S DISEASE, NEURODEGENERATIVE DISORDERS, AND TRAUMA

**DOI:** 10.1093/geroni/igad104.2746

**Published:** 2023-12-21

**Authors:** Larry Tupler, Arseniy Yashkin, Masudul Hoque, Anatoliy Yashin, Igor Akushevich, Eric Stallard

**Affiliations:** Duke University Medical Center, Durham, North Carolina, United States; Duke University, Durham, North Carolina, United States; SSRI, Duke University, Durham, North Carolina, United States; Social Science Research Institute, Duke University, Durham, North Carolina, United States; Duke University, Durham, North Carolina, United States; Duke University, Durham, North Carolina, United States

## Abstract

Alzheimer’s disease (AD) is a form of progressive dementia associated with significant personal, social, and economic burdens, making targeted interventions aimed at mitigating the effects of this condition on cognitive decline highly desirable. However, AD is but one of a range Neurodegenerative Disorders (ND) classified as dementias of aging. Furthermore, neither AD/ ND nor cognitive decline itself are monolithic: each individual disorder degrades specific aspects of cognition at differing rates. Isolating the most vulnerable facet of good cognitive health associated with each condition can lead the way to more personalized health interventions and an overall reduction of the burdens posed by AD /ND. In this paper we assess how the presence of AD and/or other ND degrade immediate (IR) and delayed (DR) recall over an extended period of time vs. the effect of non-degenerative brain trauma and AD/ND-free aging. Using Medicare-linked data from the Health and Retirement Study we found that, when compared to AD/ND-free aging, the onset of AD accelerates the decrease of IR by an overage of 0.30-0.37 points bi-annually, with similar effects on DR. Vascular dementia was associated with a 0.24-point loss of IR, but only for individuals with initially low recall capacity (≤5); those with high initial recall capacity (6+) did not experience loss of IR but lost 1.01 points of DR bi-annually. For comparison, traumatic brain injury was associated with 0.12-0.23 points decline in IR and 0.15 in DR bi-annually.

